# Overexpression of Striated Muscle Activator of Rho Signaling (STARS) Increases C2C12 Skeletal Muscle Cell Differentiation

**DOI:** 10.3389/fphys.2016.00007

**Published:** 2016-02-08

**Authors:** Marita A. Wallace, Paul A. Della Gatta, Bilal Ahmad Mir, Greg M. Kowalski, Joachim Kloehn, Malcom J. McConville, Aaron P. Russell, Séverine Lamon

**Affiliations:** ^1^Centre for Physical Activity and Nutrition Research, School of Exercise and Nutrition Sciences, Deakin UniversityBurwood, VIC, Australia; ^2^Department of Biochemistry and Molecular Biology, Bio21 Institute of Molecular Science and Biotechnology, University of MelbourneParkville, VIC, Australia

**Keywords:** skeletal muscle, proliferation, differentiation, myogenesis, regeneration

## Abstract

**Background:** Skeletal muscle growth and regeneration depend on the activation of satellite cells, which leads to myocyte proliferation, differentiation and fusion with existing muscle fibers. Skeletal muscle cell proliferation and differentiation are tightly coordinated by a continuum of molecular signaling pathways. The striated muscle activator of Rho signaling (STARS) is an actin binding protein that regulates the transcription of genes involved in muscle cell growth, structure and function via the stimulation of actin polymerization and activation of serum-response factor (SRF) signaling. STARS mediates cell proliferation in smooth and cardiac muscle models; however, whether STARS overexpression enhances cell proliferation and differentiation has not been investigated in skeletal muscle cells.

**Results:** We demonstrate for the first time that STARS overexpression enhances differentiation but not proliferation in C2C12 mouse skeletal muscle cells. Increased differentiation was associated with an increase in the gene levels of the myogenic differentiation markers *Ckm, Ckmt2* and *Myh4*, the differentiation factor *Igf2* and the myogenic regulatory factors (MRFs) *Myf5* and *Myf6*. Exposing C2C12 cells to CCG-1423, a pharmacological inhibitor of SRF preventing the nuclear translocation of its co-factor MRTF-A, had no effect on myotube differentiation rate, suggesting that STARS regulates differentiation via a MRTF-A independent mechanism.

**Conclusion:** These findings position STARS as an important regulator of skeletal muscle growth and regeneration.

## Introduction

Pre- and post- natal skeletal muscle development and regeneration of mature adult skeletal muscle require the coordinated control of myocyte proliferation and differentiation (Buckingham et al., 2003; Bentzinger et al., 2012). Each stage of muscle cell development and maturation is precisely regulated by the basic helix-loop-helix (bHLH) E-box binding protein family of myogenic regulatory factors (MRFs; Zhang et al., 1995; Kablar et al., 1997; Perry and Rudnick, 2000; Yokoyama and Asahara, 2011). Serum response factor (SRF) is a MADS box protein that interacts with MRFs to further enhance myoblast proliferation and myotube differentiation (Black and Olson, 1998; Pipes et al., 2006b; Yokoyama and Asahara, 2011). SRF regulates cytoskeletal dynamics, myogenic gene expression and myocyte proliferation and differentiation via the transcriptional control of immediate early genes such as *Fos, Lif, Junb* and *Egr1*, as well as via muscle specific genes including actin and myosin (Chai and Tarnawski, 2002; Pipes et al., 2006b; Miano et al., 2007). Attenuating SRF activity disrupts myoblast proliferation and differentiation (Soulez et al., 1996), while SRF knockdown impairs skeletal muscle development *in vivo* (Zhang et al., 2001; Li et al., 2005). Identifying the molecular factors that regulate SRF activity is therefore essential to comprehend the mechanisms underlying skeletal muscle development and regeneration.

Among the known SRF regulators, striated muscle activator of Rho signaling (STARS; also known as MS1 and ABRA) is a muscle specific actin binding protein that associates with the I-band, Z-disk and M-line of the sarcomere (Arai et al., 2002). In conjunction with RhoA, STARS increases actin polymerization, resulting in the nuclear translocation of the SRF transcriptional co-activator myocardin-related transcription factor-A (MRTF-A) and enhanced SRF transcriptional activity (Arai et al., 2002; Kuwahara et al., 2005). STARS is responsive to mechanical stress and its expression increases in rodent cardiac muscle in response to pressure overload and in rabbit smooth muscle following fluid shear-induced arteriogenesis (Mahadeva et al., 2002; Kuwahara et al., 2007; Troidl et al., 2009). In human skeletal muscle, we and others have shown that STARS is increased in response to resistance and endurance acute exercise and exercise training (Lamon et al., 2009; MacNeil et al., 2010; Wallace et al., 2011). STARS may therefore act as a sensor to transduce extracellular mechanical stress signals to activate the molecular pathways regulating muscle development, growth and regeneration. STARS is transcriptionally regulated by MEF2, MyoD, PGC-1α/ERRα and SRF, transcription factors playing multiple roles in myocyte proliferation and differentiation (Naya and Olson, 1999; Handschin et al., 2003; Kuwahara et al., 2007; Ounzain et al., 2008; Murray and Huss, 2011; Wallace et al., 2011; Chong et al., 2012). In addition, STARS can regulate its own expression via a feed-forward mechanism that requires SRF binding to the serum response element (SRE) located on the STARS promoter (Chong et al., 2012). STARS is expressed during skeletal muscle early embryonic development and its expression increases during post-natal muscle development (Arai et al., 2002; Peng et al., 2008; Chong et al., 2012). *In vitro*, the *Stars* gene is more highly expressed in terminally differentiated skeletal myotubes than in proliferating myoblasts (Ounzain et al., 2008). Overexpressing STARS in porcine smooth muscle cells and in the A10 rat vascular smooth muscle cell line (Troidl et al., 2009) accelerates proliferation, supporting a role for STARS in muscle development.

Here we test the hypothesis that STARS may play a similar role in the regulation of skeletal muscle cell proliferation and extend our investigations to myotube differentiation. We report for the first time that STARS overexpression in C2C12 mouse muscle cells enhances cell differentiation, but not proliferation, and upregulates the expression of several myogenic differentiation markers, differentiation factors and MRFs. CCG-1423, a pharmacological inhibitor of SRF that limits the nuclear translocation of MRTF-A, did not attenuate basal or STARS-induced increase in differentiation rate, suggesting that STARS regulates C2C12 cells differentiation via a MRTF-A-independent pathway.

## Materials and methods

### Construction of pFLAG-mSTARS expression vector

The cDNA for mouse STARS was subcloned into the pFLAG-CMV4 (Sigma-Aldrich, Castle Hill, NSW) mammalian expression plasmid and was designated pFLAG-mSTARS. The DNA template used to produce the mouse STARS DNA fragment was the pBluescript-mouseSTARS plasmid, which was previously cloned by our group. Polymerase chain reaction (PCR) using specifically designed primers (Geneworks, Hindmarsh, SA) was performed to amplify the mouse STARS DNA sequence and incorporate the desired restriction enzyme sites, EcoRI, and BamHI. PCR products were separated on a 1.0% agarose gel and the ~1000 base mouse STARS DNA fragment was extracted and purified using the QIAquick Gel extraction Kit (QIAGEN, Doncaster, VIC). The mouse STARS DNA fragment and pFLAG-CMV4 plasmid were digested using the EcoRI and BamHI restriction enzymes (NEB, Ipswich, MA) and subsequently ligated to produce the pFLAG-mSTARS plasmid. Amplification of pFLAG-mSTARS was achieved by transformation into 5-α Competent *E. coli* (High Efficiency) cells (NEB, Ipswich, MA) and plasmid DNA extraction and purification was performed using the QIAGEN plasmid kit (QIAGEN, Doncaster, VIC). Finally, automated sequencing (Applied Genetic Diagnostics, Parkville, VIC) was performed.

### Cell culture

C2C12 mouse myoblasts (ATCC, Manassas, VA) were maintained in complete Dulbecco's Modified Eagle's Medium (DMEM) supplemented with 10% fetal bovine serum (FBS; Life Technologies, Melbourne, Australia) at 37°C and 5% CO_2_. For differentiation experiments, when cultures approached confluence (~90% confluent), medium was changed to differentiation medium (DMEM) supplemented with 2% horse serum (HS; Life Technologies, Melbourne, Australia). Differentiation medium was replaced every 24 h.

### Transient transfection with pFLAG-mSTARS in C2C12 myoblasts

C2C12 myoblasts (1.0–2.0 × 10^4^ cells per well) were plated into six-well plates or 35 mm μ-Dishes (ibidi, Munich, Germany) 24 h before transfection. Myoblasts were transiently transfected with the pFLAG-mSTARS or pFLAG-CMV4 (Sigma-Aldrich, Castle Hill, NSW) plasmid using Lipofectamine 2000 (Life Technologies, Mulgrave, VIC). Myoblasts were exposed to transfection reagents for 24 h, following what myoblasts were incubated in complete DMEM (10% FBS) for 24 h. RNA and protein were harvested for RT-PCR gene expression and western blot analysis, respectively.

### MRTF-A/SRF transcriptional inhibition with CCG-1423 in C2C12 myoblasts

To provide proof-of concept that the pharmacological inhibitor CCG-1423 prevents MRTF-A translocation to the nucleus during all stages of C2C12 cells differentiation, 2.0 × 10^4^ or 2.0 × 10^5^ myoblasts were plated in 6 well plates or 35 mm μ-Dishes (ibidi, Munich, Germany), respectively. Myoblasts were allowed to growth for 48 h in growth medium as described above, following what the medium was replaced by serum-free DMEM supplemented with either 5 μM CCG-1423 (Merck Millipore, Kilsyth, VIC) or dimethyl sulfoxide (DMSO) at a final concentration of 0.1%. Similarly, myotubes were differentiated as described above and were serum-starved after either 48 or 96 h of differentiation. Following overnight incubation, the medium was changed to DMEM supplemented with 20% FBS containing the same concentrations of CCG-1423 or DMSO, respectively. After 1 h, the cells were prepared for immuno-localisation.

### BrdU assay

The rate of C2C12 myoblast proliferation was assessed using the colorimetric 5-bromo-2′-deoxy-uridine (BrdU) Labeling and Detection Kit III assay (Roche Applied Science, Indianapolis, USA) modified as follows. C2C12 cells were plated at a concentration of 10^4^ cells/ml in a 6-well tissue culture plate. Twenty-four hours later, myoblasts were transfected with pFLAG-mSTARS or pFLAG-CMV4 as described above, following what myoblasts were incubated in complete DMEM (10% FBS) for 24 h. Twenty-four hours before the analysis, the BrdU label reagent and CCG-1423 or DMSO treatment were added to the medium as described above. Nucleases were used to fragment DNA and BrdU incorporation into the DNA was detected using a horseradish peroxidase conjugated anti-BrdU antibody. Following the addition of the peroxidase substrate ABTS (2,2′-azino-bis, 3-ethylbenzthiazoline-6-sulphonic acid), fluorescence was detected at 405 nm.

### DNA synthesis using deuterated water

In addition to the BrdU cell proliferation assay, C2C12 myoblast proliferation was confirmed by measuring DNA synthesis using deuterated “heavy” water (^2^H_2_O). The use of the stable isotopic tracer ^2^H_2_O for measuring DNA synthesis has numerous advantages over other existing tracer methods including virtually no cell toxicity (at low ^2^H_2_O concentrations, < 10%), true precursor uniformity and greater tracing potential of the nucleotide synthesis pathways (Busch et al., 2007). C2C12 cells were prepared as described above for the BrdU assay. Twenty-four hours before the analysis, the culture medium containing 5% BSA was enriched with ^2^H_2_O at a concentration of 4% and analysis performed as detailed previously (Kloehn et al., 2015). Briefly, 2000–5000 ng of cell nucleic acid was extracted, enzymatically hydrolyzed, and dephosphorylated (Busch et al., 2007) and the released nucleosides were derivatized with O-(2,3,4,5,6-pentafluorobenzyl) hydroxylamine hydrochloride (PFBHA HCl, 25 mg/ml in water; Sigma-Aldrich, Castle Hill, NSW) at 90°C, pH 2 for 3 h. Deoxyribose sugars are released from nucleobases during oximation and their PFBHA derivative was extracted in ethyl acetate/hexane (1:1) and dried under nitrogen. The dried samples were then silylated by addition of ethyl acetate (20 μl) and N,O-bis(trimethylsilyl) trifluoroacetamide reagent (40 μl BSTFA + 1% TMCS) (Thermo Scientific, Rockford, IL) and incubated at 90°C for 30 min. The perfluorotritrimethylsilyl derivative of deoxyribose (MW: 545) was analyzed by GC/MS in positive chemical ionization mode using methane as reagent gas. The M0-M1 fragment ions [M+1-16]^+^ of deoxyribose (530–531 m/z), corresponding to the loss of CH_4_, were analyzed and the excess M1 molar enrichments (EM1 %) calculated (Busch et al., 2007; Kloehn et al., 2015).

### MRTF-A cellular localization

To determine MRTF-A cell localization, myoblasts were fixed with 4% paraformaldehyde (PFA)/PBS for 10 min after incubation with DMEM supplemented with 20% FBS or serum-free DMEM for 1 h. Cells were permeabilised in 0.1% Trition X-100/PBS for 5 min and blocked with 1% BSA/PBS for 1 h at room temperature. Cells were incubated overnight at 4°C with a MRTF-A antibody (H-140) (sc-32909; Santa Cruz Biotechnology Inc, Dallas TX) diluted 1:500 in 1% BSA/PBS and subsequently with an Alexa Fluor® 594 goat anti-rabbit IgG secondary antibody (Life Technologies, Melbourne, VIC) diluted 1:1000 and 0.1 μg/ml DAPI stain (Sigma-Aldrich, Castle Hill, NSW) in 1%BSA/PBS for 1 h at room temperature. Cell images were obtained using the Olympus Fluoview FV10i confocal laser scanning microscope with dedicated software at a 150x magnification for myoblasts and at a 120x magnification for myotubes using consistent fluorescence thresholds and exposure times. The ratio between MRTF-A fluorescence in the nuclei against total MRTF-A fluorescence in the entire cell was calculated from a minimum of 10 images per group using the Image J software (U. S. National Institutes of Health, Bethesda).

### Adenoviral infection with ADV-STARS

Adenoviral constructs containing the mouse STARS (ADV-STARS) or the control gene LacZ (ADV-LacZ) were a generous gifts from Eric N. Olson, The University of Texas Southwestern Medical Center. C2C12 myoblasts were plated in six-well tissue culture plates or 35 mm μ-Dishes in complete DMEM (10%FBS) and allowed to reach ~90% confluence. Cells were infected with a multiplicity of infection (MOI) of 100 for both the ADV-STARS and the ADV-LacZ in differentiation medium (DMEM) supplemented with 2% HS. Cells were allowed to differentiate for 5 days and medium was replaced every 24 h. RNA and protein were harvested at day 1, 3, and 5 of the differentiation process for RT-PCR gene expression and western blot analysis.

### Quantification of myotube fusion

Following 5 days of differentiation, C2C12 myotubes were fixed with 4% PFA/PBS for 10 min. Fixed cells were permeabilised in 0.1% Trition X-100/PBS and blocked with 1% BSA/PBS for 1 h at room temperature. Cells were incubated overnight at 4°C with a primary antibody against sarcomeric myosin (MF-20, Developmental Studies Hybridoma Bank) diluted 1:50 in 1% BSA/PBS. Following this, the cells were incubated with an Alexa Fluor® 488 goat anti-mouse IgG secondary antibody (Life Technologies, Melbourne, VIC) diluted 1:1000 and 0.1 μg/ml DAPI stain (Sigma-Aldrich, Castle Hill, NSW) in 1% BSA/PBS for 1 h at room temperature. Cell images were obtained using the Olympus Fluoview FV10i confocal laser scanning microscope with dedicated software at a 20x magnification. To assess myogenic differentiation, the number of DAPI-stained nuclei within the sarcomeric myosin positive myotubes (multinucleated cells, i.e., ≥2 nuclei) was determined and expressed as a percentage of the total number of nuclei analyzed per image. A minimum of 40 images per group (from two separate experiments) were analyzed using the ImageJ Software (National Institutes of Health, Bethesda, MA).

### MRTF-A/SRF transcriptional inhibition with CCG-1423 in C2C12 myotubes

Confluent (~90%) C2C12 myoblasts were infected with either the ADV-STARS or the vector control adenovirus (ADV-LacZ) as described above. The cells were then treated with either 5 μM CCG-1423 (Merck Millipore, Kilsyth, VIC) or control vehicle (DMSO) in DMEM (2%HS) for 24 h. The final DMSO concentration in both treatments was 0.1%. Cells were allowed to differentiate for 5 days and treatments were replaced every 24 h.

### Cytotoxicity assay

Cytotoxicity of the SRF inhibitor CCG-1423 was assessed using the CellToxTM Green Cytotoxicity Assay (Promega, Auburn, VIC) according to the manufacturer's instructions. Briefly, C2C12 myoblasts were seeded at a density of 0.5 × 10^4^ cells per well in back-walled 96-well tissue culture plates (ibidi, Munich, Germany) and grown in growth media for 48 h. Myoblasts were then treated with 5 μM CCG-1423 (Merck Millipore, Kilsyth, VIC) or 0.1% DMSO in serum free media for 24 h. C2C12 myotubes were differentiated as described above. After 96 h of differentiation, the cells were treated with 5 μM CCG-1423 (Merck Millipore, Kilsyth, VIC) or 0.1% DMSO in serum free media for 24 h and fluorescence was measured at 485–500 nmEX/520–530 nmEM using a Synergy 2 multi-mode microplate reader (Biotek, Currumbin, QLD) to assess cell viability.

### RNA extraction

Total RNA was extracted using the Tri-Reagent® Solution (Ambion Inc, Austin TX) according to the manufacturer's protocol. First-strand cDNA was generated from 1 to 2 μg RNA in 20 μL reaction buffer using the High Capacity RNA-to-cDNA kit (Applied Biosystems, Forster City, CA) according to the manufacturer's protocol. Before diluting cDNA, 1 μL ribonuclease H (Rnase H; Life Technolgies, Mulgrave, VIC) was added to each sample and incubated at 37°C for 30 min. Following this, cDNA was diluted in NFW to 5 ng/μL and stored at −20°C until further analysis.

### Real time quantitative PCR

Real-time PCR was carried out using the Stratagene MX3000 PCR system (Agilent Technologies, Santa Clara CA) to measure the mRNA levels of genes of interest. To compensate for variations in input RNA amounts and efficiency of the reverse transcription, data was normalized to single-stranded DNA (ssDNA) content that was determined using the Quanti-iT OliGreen ssDNA Assay Kit (Molecular Probes, Eugene, OR). RT-PCR primer details for the mouse genes are provided in Table 1.

**Table 1:** **Details of mouse primers used for RT-PCR analysis**.

**Gene**	**GenBank accession number**	**Forward Primer (5′–3′)**	**Reverse primer (5′–3′)**
*Ckm*	NM_007710.2	GCGCCGTGTTCGACATC	GACTGGCCCTTTTCCAGCTT
*Ckmt2*	NM_198415.2	ACGCACTGGCCGAAGCATCC	GCCAGATCGCCCTTCAGGCC
*Egr1*	NM_007913.5	GAGCGAACAACCCTATGAGC	AGGCCACTGACTAGGCTGAA
*Fos*	NM_010234.2	CACTCCAAGCGGAGACAGAT	TGGGCTGCCAAAATAAACTC
*Igf1*	NM_010512.4	GCTCTGCTTGCTCACCTTCAC	CCTCCGTCCACACACGAACT
*Igf2*	NC_000073.6	CGCTTCAGTTTGTCTGTTCG	GGAAGTACGGCCTGAGAGGTA
*JunB*	NM_008416.3	TTGCGGACGGTTTTGTCA	CGTCACGTGGTTCATCTTGTG
*Lif*	NM_008501.2	GGCAACCTCATGAACCAGAT	ACCATCCGATACAGCTCCAC
*Myh4*	NM_010855.2	ACAGACTAAAGTGAAAGCC	CTCTCAACAGAAAGATGGAT
*Myf5*	NM_008656.5	CACCACAACCAACCCTAACCA	ACTCTCAATGTAGCGGATTGC
*Myf6*	NC_000076.6	GGTACCCTATCCCCTTGCCA	GGGAGTTTGCGTTCCTCTGA
*Srf*	NM_020493.2	ACGACCTTCAGCAAGAGGAA	AAGCCAGTGGCACTCATTCT
*Stars*	NM_175456.4	TCAAACGCCCCTTGCTCTC	CGTGTTCATCGGCCCACT

### Protein extraction

Total protein was extracted using 1x RIPA buffer (Millipore, North Ryde, NSW) with 1 μL/mL protease inhibitor cocktail (Sigma-Aldrich, Castle Hill, NSW) and 10 μL/mL Halt Phosphatase Inhibitor Single-Use Cocktail (Thermo Scientific, Rockford, IL). Total protein content was determined using the BCA Protein Assay Kit (Pierce Biotechnology, Rockford, IL) according to the manufacturer's instructions.

### Western blotting

Electrophoresis and protein transfer were performed using the XCell Surelock Novex Mini- Cell (Invitrogen, Carlsbad, CA) system. Protein lysates were separated by SDS-PAGE using pre-cast NuPAGE® Novex 4–12% Bis-Tris gels (Invitrogen, Carlsbad, CA) and transferred to a PVDF membrane (Millipore, Billerica, MA). Membranes were then incubated at room temperature for 1 h in blocking buffer supplemented with 5% BSA/PBS. After blocking, membranes were incubated overnight at 4°C with the following primary antibodies diluted 1:1000 in 5% BSA/PBS: STARS (Institute of Medical and Veterinary Science, Adelaide SA); Myosin, Sarcomeric (MF-20, Developmental Studies Hybridoma Bank, Iowa City IA); SRF (Santa Cruz Biotechnology, Dallas, TX (sc-335)). Following washing the membranes were incubated for 1 h with the corresponding infrared dye-conjugated secondary antibodies (Thermo Fisher Scientific, Inc, Rockford, IL). After washing, the specific proteins were revealed using the Odyssey Imaging System (LI-COR, Lincoln, NE, USA). GAPDH (G8795, Sigma-Aldrich, Castle Hill, NSW) was used to control for protein loading and individual protein band optical densities were determined using the Odyssey Infrared Imaging System software.

### Statistical analyses

All data are reported as mean ± SEM. To ensure homogeneity of variance, all data was log10-transformed and analyses were conducted on these transformed scales. Overexpression experiments in proliferating C2C12 myoblasts were analyzed using either a two-tailed unpaired *t*-test or a one-way analysis of variance (ANOVA). For every other analysis, a mixed-model two-way ANOVA or a general ANOVA were used to compare group means using GenStat v16. Diagnostic plots of residuals and fitted values were checked to ensure homogeneity of variance (a key assumption for ANOVA). The least significant difference (LSD) test was used to compare pairs of means. The significance levels for both the *F*-tests in the ANOVA and the LSD tests were set at *p* < 0.05. Note that the statistical significance reported in the figures is based on analysis of the transformed data but the reported means ± S.E.M. are on the original (untransformed) scale.

## Results

### Endogenous expression of STARS during proliferation and differentiation of C2C12 myocytes

**Figure 1:**
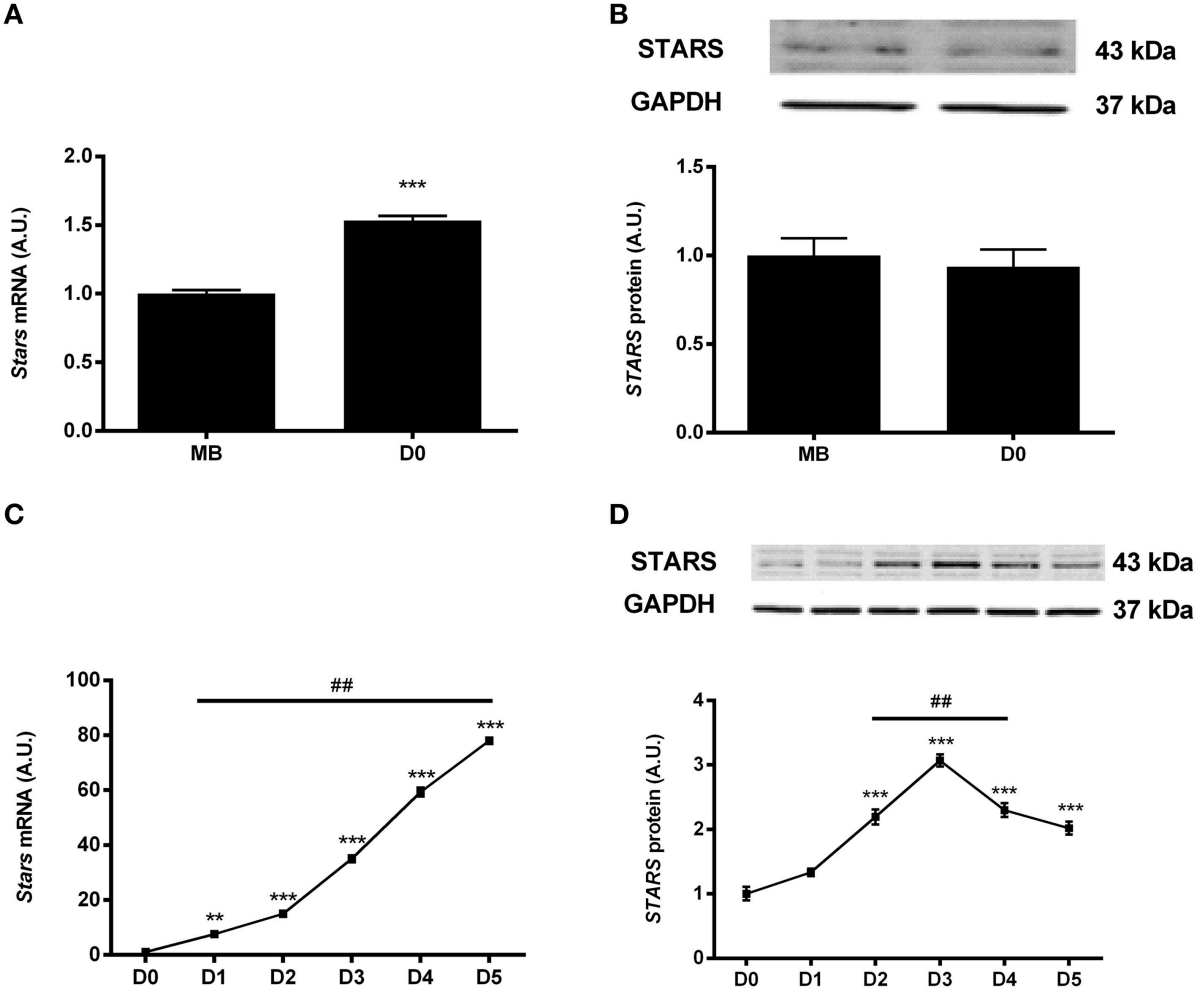
**STARS mRNA and protein expression levels were respectively measured by real-time qPCR and Western blot during C2C12 cells proliferation and differentiation**. STARS mRNA **(A)** but not protein **(B)** levels increase in confluent myoblasts (D0; prior to adding differentiation media) when compared to sub confluent myoblasts (MB). ^***^ different from MB, *p* < 0.001. STARS mRNA **(C)** and protein **(D)** levels increase during myotube differentiation. ^**^ different from D0, *p* < 0.01, ^***^ different from D0, *p* < 0.001, ^##^different from precedent day, *p* < 0.01.

*Stars* mRNA expression increased by 1.5-fold in confluent myoblasts when compared to sub-confluent myoblasts (*p* < 0.001; Figure 1A); however, there was no significant increase in *STARS* protein expression during myoblast proliferation (Figure 1B). Supplementary Figure 1 displays the full western blot images of all immunoblot pictures presented in this manuscript. *Stars* mRNA levels gradually increased during myotube differentiation to reach a 80-fold increase by differentiation day 5 when compared to proliferating myoblasts (Figure 1C). When compared to D0, *STARS* protein levels concomitantly increased by 2- to 3-fold between differentiation days 2 and 5 (Figure 1D).

### The effect of STARS overexpression on SRF-target genes and C2C12 myoblast proliferation

The overexpression of STARS in proliferating C2C12 myoblasts using a pFLAG-mSTARS plasmid (STARS) resulted in a substantial increase in *Stars* mRNA levels (*p* < 0.001) paralleled by a 15-fold increase in *STARS* protein levels (*p* < 0.001) when compared to C2C12 myoblasts transfected with a pFLAG-CMV4 plasmid (control) (Supplementary Figures 2A,B).

**Figure 2:**
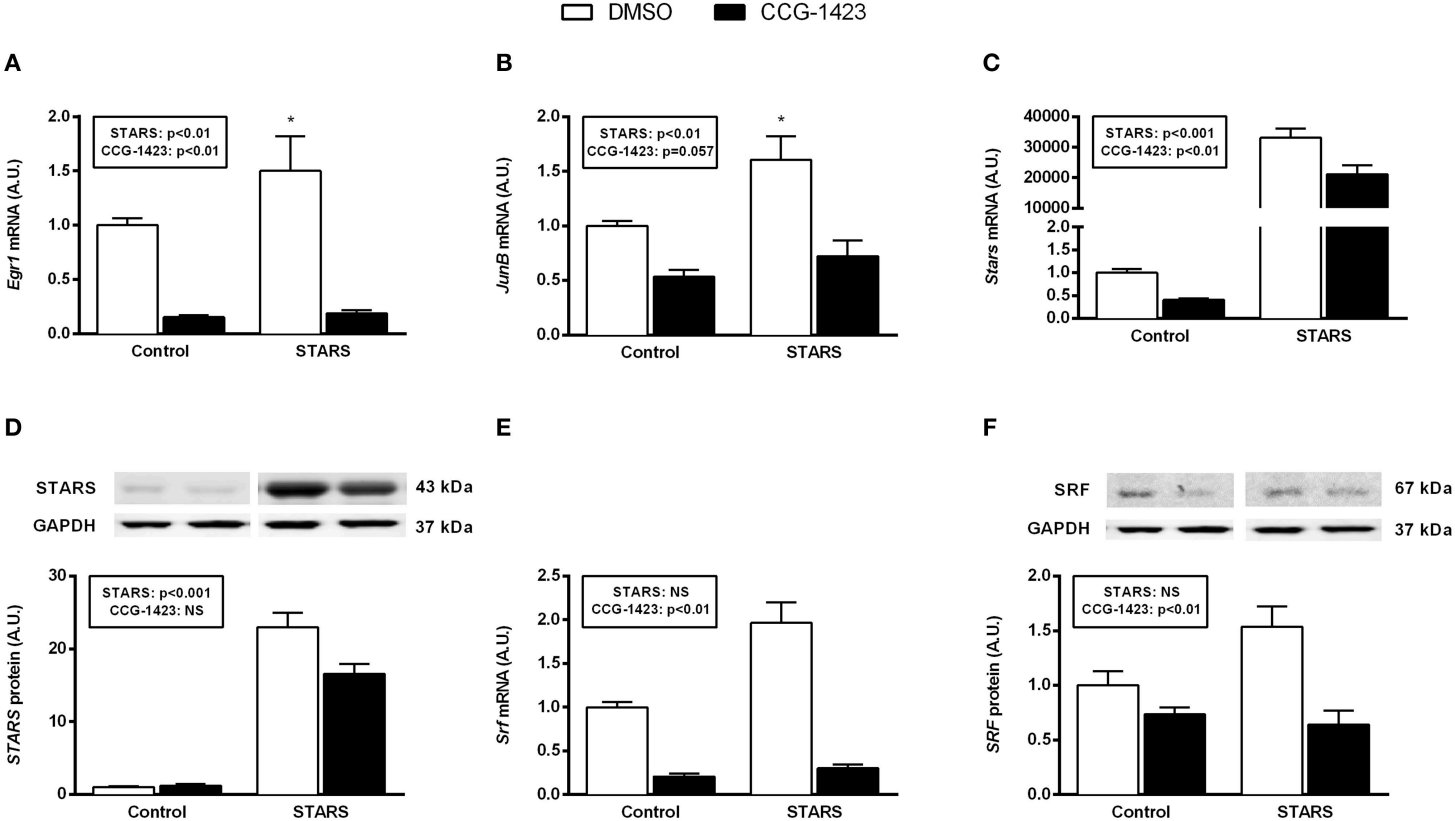
**Effect of STARS overexpression and inhibition of MRTF-A translocation on known SRF target genes (A,B), STARS mRNA (C) and protein (D), and SRF mRNA (E) and protein (F)**. mRNA and protein expression levels were respectively measured by real-time qPCR and Western blot. Inhibition of MRTF-A translocation by CCG-1423 treatment decreases the mRNA levels of *Egr-1* **(A)**, *Junb* **(B)**, *Stars* **(C)**, *Srf* gene **(E)** and SRF protein levels **(F)**. It has no effect on STARS protein levels **(D)**. In **(A)** and **(B)**, *post-hoc* tests revealed significant differences when comparing the Control DMSO to the STARS DMSO group (^*^ different from Control DMSO, *p* < 0.05). DMSO, dimethyl sulfoxide vehicle control; CCG-1423, SRF inhibitor.

**Figure 3:**
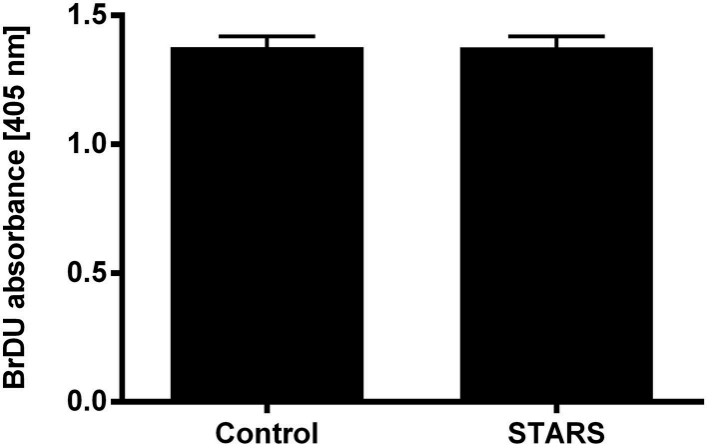
**STARS overexpression has no effect on C2C12 myoblast proliferation**. Absorbance measured at 405 nm following BrDU incorporation assay.

Immediate early genes function as early regulators of cell growth. In C2C12 myoblasts overexpressing STARS, the expression levels of the known SRF-regulated immediate early genes *Egr1* and *Junb* increased when compared to control myoblasts (Figures 2A,B; compare white bars only). There was no effect of STARS on other early genes including *Fos, Igf1* and *Lif* (data not shown). Analysis of both BrDU and deuterium incorporation assay did not reveal any difference in myoblast DNA synthesis with STARS overexpression. Thus, proliferation rates with STARS overexpression were unaltered (Figure 3 and Supplementary Figure 2C).

### The effect of SRF inhibitor CCG-1423 in C2C12 myoblasts overexpressing STARS

**Figure 4:**
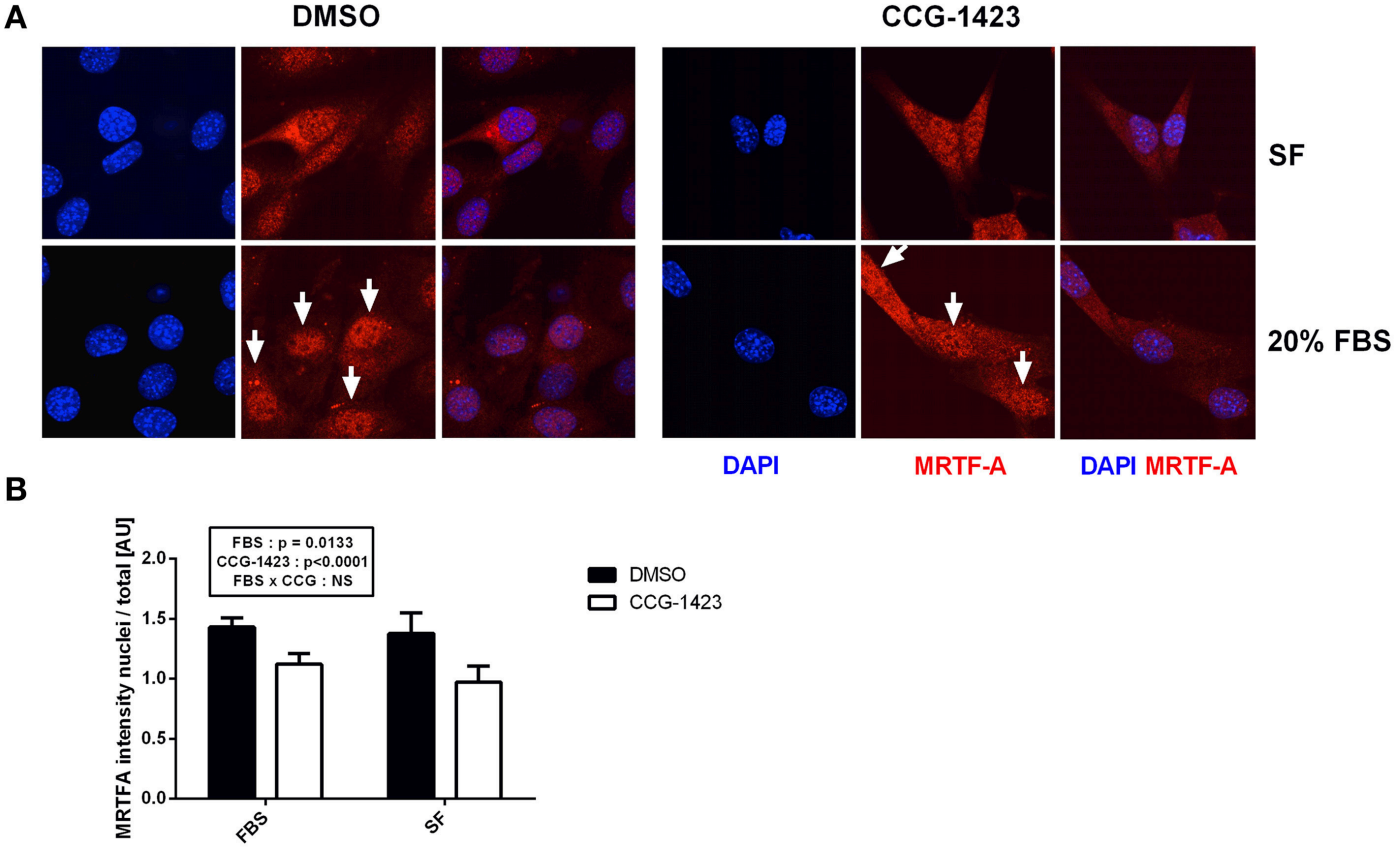
**MRTF-A cellular localization following CCG-1234 treatment in serum-stimulated (20% FBS) and serum-free (SF) C2C12 myoblasts**. Immunolocalization of MRTF-A **(A)**. Blue, DAPI staining; Red, MRTF-A; DMSO, dimethyl sulfoxide vehicle control; CCG-1423, SRF inhibitor. In the FBS treated group, arrows point toward the nuclei where MRTF-A presence is high (DMSO) or attenuated by CCG-1423 treatment (CCG-1423). MRTF-A intensity in the nucleus divided by total MRTF-A intensity **(B)**. CCG-1423 treatment prevented MRTF-A nuclear translocation in both serum-stimulated (FBS) and serum-free (SF) C2C12 myoblasts.

Pharmacological SRF inhibitor CCG-1423 blocks the nuclear translocation of MRTF-A and therefore partially inhibits SRF activation. We aimed to determine if MRTF-A translocation was required for the STARS-induced increase in SRF target gene expression and if STARS overexpression was able to counteract the potential negative effect of the inhibitor. CCG-1423 treatment had no cytotoxic effect on C2C12 myoblasts when compared to DMSO treatment or to untreated myoblasts (Supplementary Figure 2D). In C2C12 myoblasts, MRTF-A is localized within the cytoplasm and the nucleus under serum-free and serum stimulated (20% FBS) conditions. Following CCG-1423 treatment, we observed a decrease in MRTF-A presence in the nucleus in both conditions (*p* < 0.0001), providing proof-of-concept that CCG-1423 efficiently attenuates MRTF-A nuclear translocation in myoblasts (Figures 4A,B).

The expression levels of the SRF immediate early genes *Egr1* and *Junb* were reduced following CCG-1423 treatment (main effect for treatment, *p* < 0.01 and *p* = 0.06, respectively); however, this decrease could not be prevented by STARS overexpression (Figures 2A,B). CCG-1423 treatment reduced endogenous *Stars* mRNA expression in control myoblasts (0.4-fold; *p* < 0.01) and attenuated the increase in exogenous *Stars* in myoblasts transfected with the pFLAG-mSTARS plasmid (0.65-fold; *p* < 0.01; Figure 2C). These observations were paralleled by a significant decrease (*p* < 0.01) in exogenous but not in endogenous *STARS* protein expression levels (Figure 2D). SRF is known to regulate its own transcription. While *Srf* mRNA and *SRF* protein expression levels were not influenced by STARS overexpression, they were both significantly decreased following CCG-1423 treatment (*p* < 0.01; Figures 2E,F).

### The effect of STARS overexpression on C2C12 myotube differentiation

**Figure 5:**
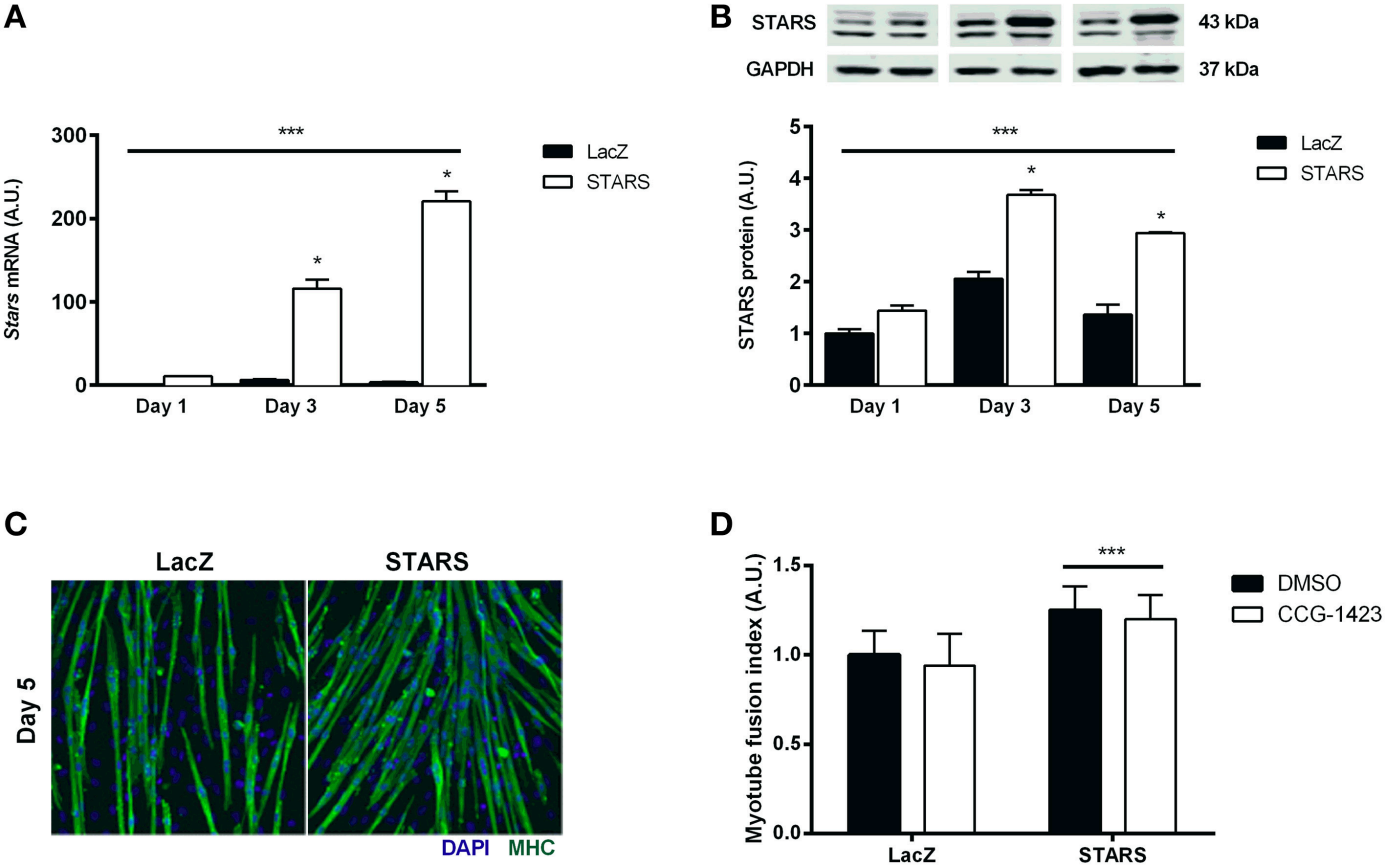
**STARS mRNA and protein levels gradually increase following overexpression in C2C12 myotubes and STARS overexpression accelerates myotube differentiation**. mRNA and protein expression levels were respectively measured by real-time qPCR and Western blot. STARS mRNA **(A)** and protein **(B)** levels increase during 5 days of myotube differentiation. ^***^ Main effect of STARS overexpression, *p* < 0.001. ^*^ Significantly different from LacZ at the same time point, *p* < 0.05. Immunofluorescent staining of MHC (green) and nuclei (blue) in differentiated myotubes at day 5 **(C)**. Myotube fusion index at differentiation day 5 increases with STARS overexpression but is not influenced by CCG-1423 treatment **(D)**. ^***^ Main effect of STARS overexpression, *p* < 0.001.

STARS overexpression in C2C12 myotubes resulted in a gradual increase in STARS mRNA and protein levels at differentiation days 3 and 5 (*p* < 0.05) when compared to control cells (Figures 5A,B). An increase in differentiation rate following STARS overexpression was assessed both visually (Figure 5C) and by the calculation of the myotube fusion index that was higher in myotubes overexpressing STARS than in control myotubes (*p* < 0.001; Figure 5D). In line with this result, STARS overexpression during differentiation increased the mRNA levels of the differentiation markers *Ckmt2, Ckm* and *Myh4* (Mhc-IIb), of the differentiation factor *Igf2* and of the MRFs *Myf5* and *Myf6* (all *p* < 0.001) (**Figures 7A–E**), but not of MyoG mRNA or protein levels (data not shown) when compared to control myotubes.

### The effect of SRF inhibitor CCG-1423 in C2C12 myotubes overexpressing STARS

**Figure 6:**
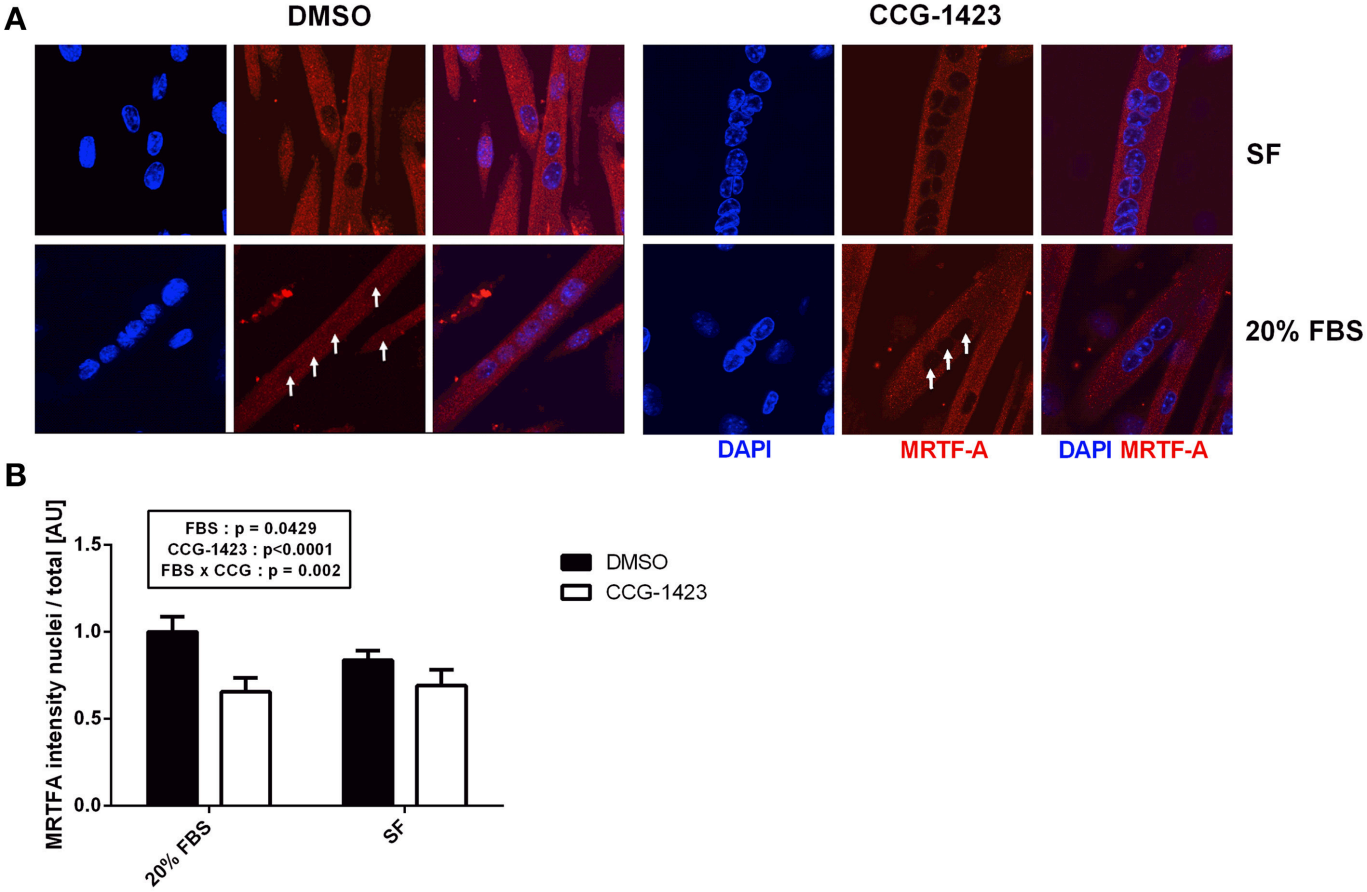
**MRTF-A cellular localization following CCG-1234 treatment in serum-stimulated (20% FBS) and serum-free (SF) C2C12 myotubes at differentiation day 3**. Immunolocalization of MRTF-A **(A)**. Blue, DAPI staining; Red, MRTF-A; DMSO, dimethyl sulfoxide vehicle control; CCG-1423, SRF inhibitor. In the FBS treated group, arrows point toward the nuclei where MRTF-A presence is high (DMSO) or attenuated by CCG-1423 treatment (CCG-1423). MRTF-A intensity in the nucleus divided by total MRTF-A intensity **(B)**. Serum stimulation (FBS) increased MRTF-A presence in the nucleus when compared to serum-free (SF) conditions. CCG-1423 treatment prevented MRTF-A nuclear translocation in both serum-stimulated and serum-free C2C12 myotubes.

**Figure 7:**
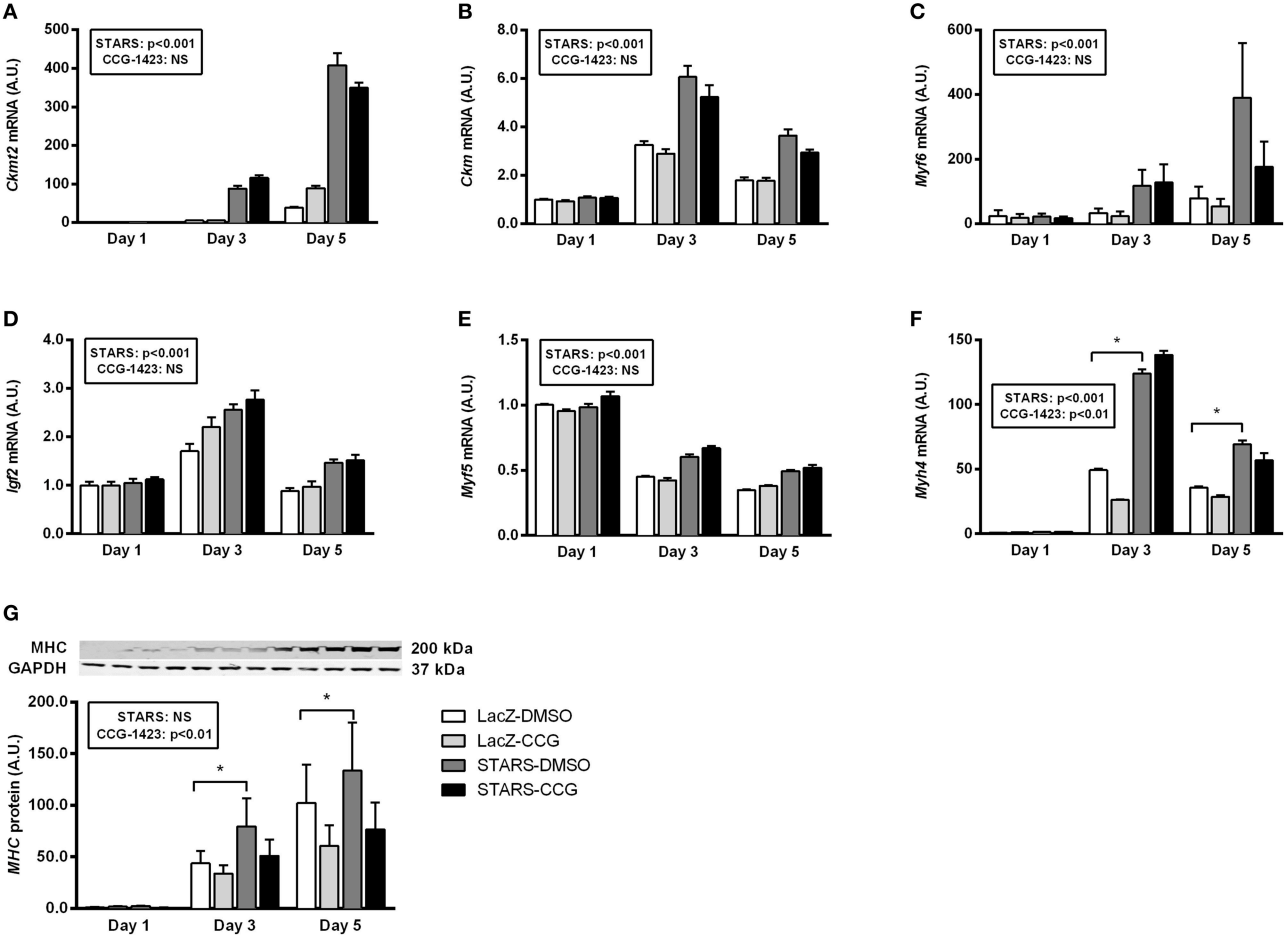
**The effect of STARS overexpression and inhibition of MRTF-A translocation on C2C12 myotube differentiation markers**. mRNA and protein expression levels were respectively measured by real-time qPCR and Western blot. STARS overexpression increases the mRNA levels of *Ckmt2* **(A)**, *Ckm* **(B)**, *Myf6* **(C)**, *Igf2* **(D)**, *Myf5* **(E)** and *Myh4* (Mhc-IIb) **(F)** when compared to control myotubes (main effect of STARS, all *p* < 0.001). In addition, there was a main effect of CCG-1423 treatment on the expression levels of *Myh4* (Mhc-IIb) mRNA **(F)** and MHC protein **(G)** (main effect of CCG-1423 treatement, both *p* < 0.01). In **(F)** and **(G)**, *post-hoc* tests revealed that CCG-1423 treated group was different from DMSO treated group at Day 3 and Day 5 (^*^ different from the DMSO group at the same time point, *p* < 0.05).

CCG-1423 treatment demonstrated no cytotoxic effect on C2C12 myotubes when compared to DMSO treatment or to untreated myotubes (Supplementary Figure 2E). Serum stimulation (20% FBS) increased nuclear MRTF-A levels in C2C12 myotubes at differentiation day 3 (*p* < 0.05) (Figures 6A,B) but not at differentiation day 5 (Supplementary Figures 2H,G). At day 3, this increase was attenuated when the cells were treated with CCG-1423 (*p* < 0.0001), although MRTF-A presence in the nucleus also decreased to a smaller extend under serum-free conditions (Figure 6B). CCG-1423 treatment did not influence myotube differentiation rate in both control myotubes and myotubes overexpressing STARS (Figure 5D). CCG-1423 had no effect on *Ckmt2, Ckm, Myf5, Myf6, Igf2* (Figures 7A–E) and *Myog, Stars* or *Srf* expression during differentiation (data not shown). On the other hand, *post-hoc* analyses revealed that in addition to a main treatment effect, CCG-1423 decreased *Myh4* gene expression and *MHC* protein levels at Day 3 and Day 5 in both groups (Figures 7F,G).

## Discussion

Skeletal muscle growth and regeneration is controlled by intracellular signals regulating myoblast proliferation and differentiation into mature multinucleated myotubes (Chambers and McDermott, 1996). The present study investigated whether STARS overexpression enhances the proliferation and differentiation of C2C12 muscle cells. We demonstrate for the first time that overexpressing STARS at the onset of myoblast differentiation enhances skeletal muscle cell differentiation. The increase in myotube fusion index was paralleled by increases in the mRNA levels of the differentiation markers *Ckm, Ckmt2, Myh4* (Mhc-IIb), of the differentiation factor *Igf2* and of the MRFs *Myf5* and *Myf6* (MRF4). On the other hand, STARS overexpression had no effect on myoblast proliferation, although it increased the mRNA levels of the SRF immediate early genes *Egr1* and *Junb*. While the pharmacological inhibitor of MRTF-A translocation CCG-1423 had a negative effect on SRF target gene expression, myotube differentiation rate was not attenuated.

The expression pattern of the STARS gene and protein during skeletal muscle cell proliferation and differentiation has only been partially investigated. *In vivo, Stars* starts to be expressed during the early embryonic development of skeletal muscle and continues to increase during post-natal muscle development (Peng et al., 2008). We observed an increase in *Stars* gene expression during myoblast proliferation and a further gradual increase during myotube differentiation, confirming previous observations made in C2C12 cells (Ounzain et al., 2008). For the first time we extended these observations to the expression levels of the *STARS* protein, which significantly increased between differentiation day 2 and 5. Together with our previous findings that STARS is required for myotube survival (Wallace and Russell, 2013), these observations suggest that increasing levels of STARS are necessary for optimal myocyte development.

STARS activates SRF-dependant transcription by directly stimulating the promoters of genes regulated by SRF, such as *Tagln2* (formerly Sm22-α) and *Acta1* (formerly α-actin) (Arai et al., 2002). In skeletal muscle, SRF regulates cell growth via the transcription of immediate early genes such as *Fos, Lif, Junb* and *Egr1* (Chai and Tarnawski, 2002; Pipes et al., 2006a; Miano et al., 2007); genes that enhance myoblast proliferation in L6 and C2C12 cells (Rahm and Sejersen, 1990; Chalaux et al., 1998). In rat H9c2 cells, overexpressing STARS significantly increased the expression levels of several SRF target genes, including *Junb, Lif* and *IL6* (Koekemoer et al., 2009; Chong et al., 2012). Here we report that STARS overexpression in skeletal muscle myoblasts increased the mRNA levels of some, but not all immediate early genes. STARS is however not the only signaling pathway able to activate SRF transcriptional activity. The MAPK pathway typically regulates the expression of SRF immediate early genes (Posern and Treisman, 2006; Sun et al., 2006; Muehlich et al., 2008) and STARS and MAPK signaling are believed to play exclusive but complementary roles in SRF transcriptional activation (Janknecht et al., 1993; Hill et al., 1995; Sotiropoulos et al., 1999; Gineitis and Treisman, 2001; Copeland and Treisman, 2002; Murai and Treisman, 2002). Our results support the observation (Selvaraj and Prywes, 2004) that the STARS pathway also influences SRF control of a subset of immediate early target genes, including *Egr1* and *Junb*. Despite the positive regulation of the early regulators of gene growth *Egr1* and *Junb*, STARS overexpression did not induce an increase in skeletal muscle cell proliferation. While STARS increases proliferation in porcine smooth muscle cells and in the A10 rat vascular smooth muscle cell line (Troidl et al., 2009), it has no effect on the proliferation of porcine aortic endothelial cells (Troidl et al., 2009) or of the H9c2 rat cardiac cell line (Koekemoer et al., 2009), suggesting cell- and species-specific influences.

We then investigated whether the STARS-induced increase in several SRF target genes was a direct consequence of STARS facilitating the nuclear translocation of the SRF transcriptional co-activator MRTF-A. The pharmacological SRF inhibitor CCG-1423 inhibits MRTF-A co-activation of SRF transcriptional activity by preventing MRTF-A nuclear translocation. In H9c2 rat muscle cells, chromatin immunoprecipitation showed that CCG-1423 completely blocked STARS proximal reporter activity (Chong et al., 2012). In the present study, CCG-1423 treatment resulted in a significant decrease in the mRNA expression levels of the SRF target genes *Egr1* and *Junb*. This effect was independent of STARS expression levels, suggesting that supra-physiological levels of STARS in myoblasts are not sufficient to counteract the negative effect of CCG-1423. The effect of STARS on known SRF target gene expression may therefore not be via their direct regulation by STARS, but via STARS upstream regulation of the MRTF-A/SRF axis, supporting observations made in NIH 3T3 cells (Kuwahara et al., 2005). Of interest, one study showed that MRTF-A expression in pulmonary fibromyoblasts was SRF-dependant and was inhibited by CCG-1423 treatment (Sandbo et al., 2011); findings that were not observed in C2C12 myoblasts in the present study. Finally, CCG-1423 treatment reduced *Stars* and *Srf* mRNA levels and *SRF* protein levels, supporting the idea that (1) SRF can positively regulate its own transcription (Miano et al., 2007) and (2) STARS can also regulate its own transcription, which may require SRF binding to the SRE located on the STARS promoter; a binding that is completely prevented by the use of the CCG-1423 inhibitor (Chong et al., 2012).

Overexpressing STARS at the onset of myoblast differentiation enhanced myogenic differentiation in skeletal muscle cells as indicated by an increased fusion index and elevated levels of several known makers of myotube differentiation including creatine kinase and myosin heavy chain (Chamberlain et al., 1985; Brown et al., 2012) as well as the differentiation factor *Igf2* (Florini et al., 1991; Magri et al., 1991) and the MRFs *Myf5* and *Myf6* but not *MyoG.* We showed that CCG-1423 prevented MRTF-A translocation to the nucleus in differentiating myotubes; however CCG-1423 treatment did not influence myotube differentiation in control cells or in STARS overexpressing cells. Nevertheless, it had a limited effect on some of the differentiation markers and caused a reduction in the expression levels of the *Ckm* gene and *MHC* protein at differentiation day 3 and day 5 in both control myotubes and myotubes overexpressing STARS. The *Ckm* gene is a SRF target gene possessing a CArG box that functions as a SRF-binding site in its cis-regulatory region (Vincent et al., 1993). Reduced SRF transcriptional activity negatively regulates the muscle creatine kinase (*Ckm*) gene and mouse muscle lacking SRF display very low levels of *Ckm* (Charvet et al., 2006). As CCG-1423 did not prevent MRTF-A translocation in myotubes at differentiation day 5, it is likely that the observed effects on myosin heavy chain gene and protein expression levels were carried on from an earlier time point. Altogether, these results support the hypothesis that skeletal muscle cell differentiation is marginally under the control of the MRTF-A/SRF transcriptional pathway and suggest the existence of a mechanism that functions in parallel and/or complementary to MRTF-A.

It is known that STARS influences SRF-mediated gene transcription via a mechanism involving RhoA signaling (Arai et al., 2002); a pathway that is essential for the differentiation of smooth, cardiac and skeletal muscle cells (Aoki et al., 1998; Du et al., 2004). The precise mechanism underlying the STARS–RhoA interaction is unknown. However, in non-muscle cells, STARS and RhoA can act independent of each other and individually enhance SRF transcriptional activity (Arai et al., 2002), although their combined overexpression augments this effect. Our results indicate that STARS plays an essential role in the differentiation from myoblasts to mature myotubes; to which extent this process is mediated by RhoA is unknown. Another hypothesis relies on observations reporting that Z disc proteins, such as calsarcin-1, can negatively regulate calcineurin/NFAT (nuclear factor of activated T cells) signaling in cardiac tissue (Frey et al., 2004; Gautel, 2008). Calcineurin plays an important regulatory role in skeletal muscle remodeling (Ryder et al., 2005) via the activation of transduction pathways involving calcium–calmodulin-dependent protein kinase IV (CaMKIV; Wu et al., 2002), MEF-2 and PGC-1α (Lin et al., 2002). STARS may activate a calcium-dependent regulatory pathway that assists with cell differentiation, however this has not been experimentally validated. In both cases, the lack of effect of CCG-1423 treatment on myotube fusion index suggests that MRTF-A would not be an obligatory part of these mechanisms.

## Conclusion

In conclusion, we show for the first time that STARS overexpression enhances the differentiation process in mouse C2C12 muscle cells. Overexpression of STARS in proliferating C2C12 myoblasts was associated with increased expression of SRF target genes involved in muscle cell growth but did not lead to increased proliferation. STARS overexpression in differentiating myotubes was associated with increased expression of typical and muscle-specific differentiation genes associated with cell structure and metabolism. STARS-induced increase in myotube differentiation was not influenced by CCG-1423 treatment, suggesting that STARS-mediated control of differentiation may also be via an MRTF-A/SRF independent mechanism. Overall, these *in vitro* findings position STARS as an important potential regulator of skeletal muscle growth and regeneration and this now requires experimental *in vivo* validation in an appropriate mouse model.

## Author contributions

Designed the study: MW, AR, SL. Completed the experiments: MW, PD, BA, GK, JK, SL. Analyzed the data: MW, PG, GK, SL. Wrote and reviewed the manuscript: all authors. Funded the study: MM, AR, SL.

### Conflict of interest statement

The authors declare that the research was conducted in the absence of any commercial or financial relationships that could be construed as a potential conflict of interest.

## Supplementary material

Supplementary Figure 1 **Full western blot images of all immunoblot pictures presented in the manuscript**.

Supplementary Figure 2 **(A)** STARS mRNA levels following STARS overexpression in C2C12 myoblasts. **(B)** STARS protein levels following STARS overexpression in C2C12 myoblasts. **(C)** ^2^H Enrichment expressed as a percentage of EM1 following deuterium incorporation assay. **(D)** Absorbance measured at 485 nm following a cytotoxicity assay in C2C12 myoblasts. **(E)** Absorbance measured at 485 nm following a cytotoxicity assay in C2C12 myotubes. **(H)** MRTF-A cellular localization following CCG-1234 treatment in serum-stimulated (20% FBS) and serum-free (SF) C2C12 myotubes at differentiation day 5. Immunolocalization of MRTF-A. Blue, DAPI staining; Red, MRTF-A; DMSO, dimethyl sulfoxide vehicle control; CCG-1423, SRF inhibitor. **(G)** MRTF-A intensity in the nucleus divided by total MRTF-A intensity in C2C12 myotubes at differentiation day 5.
