# Supplementary material for: Overexpression of Striated Muscle Activator of Rho Signaling (STARS) Increases C2C12 Skeletal Muscle Cell Differentiation
Source: Front Physiol. 2016 Feb 8;7:7. doi: 10.3389/fphys.2016.00007 (PMC4745265; doi:10.3389/fphys.2016.00007)

Supp 2B

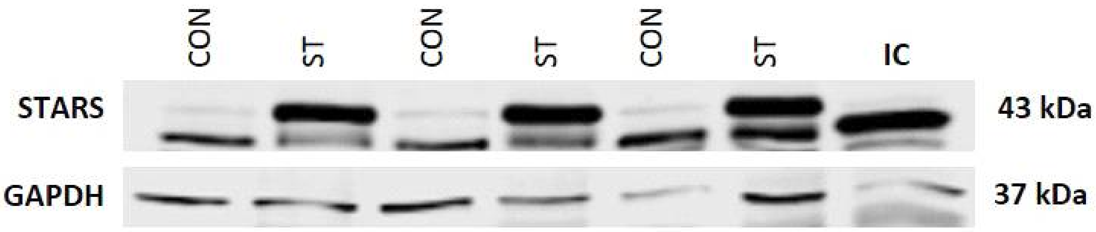

3D

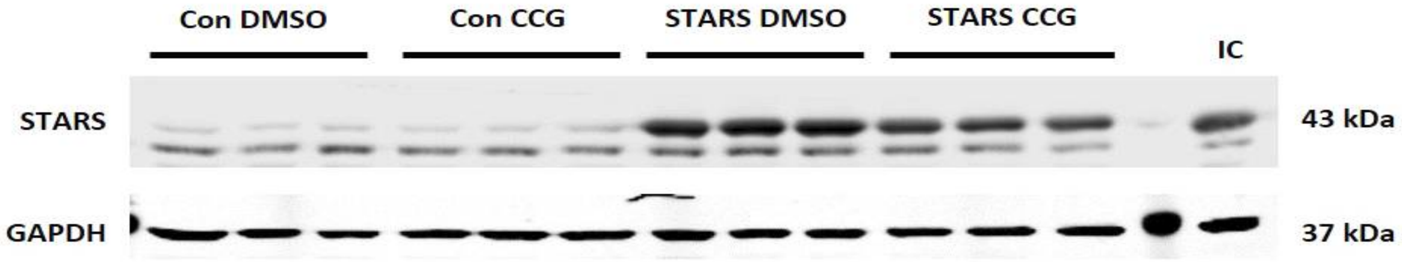

3F

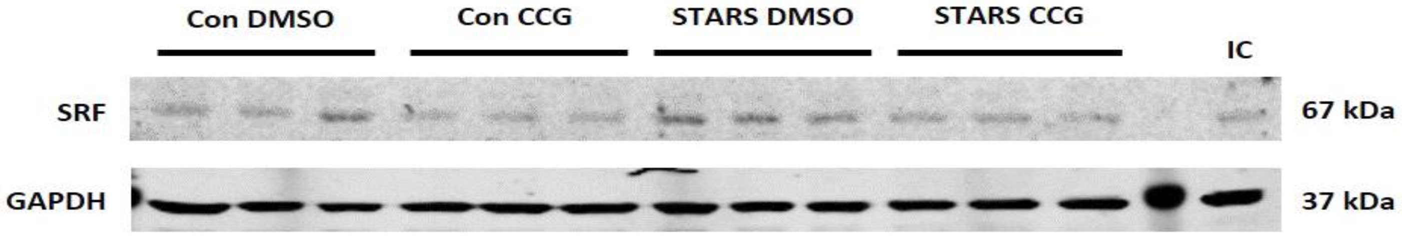

5B

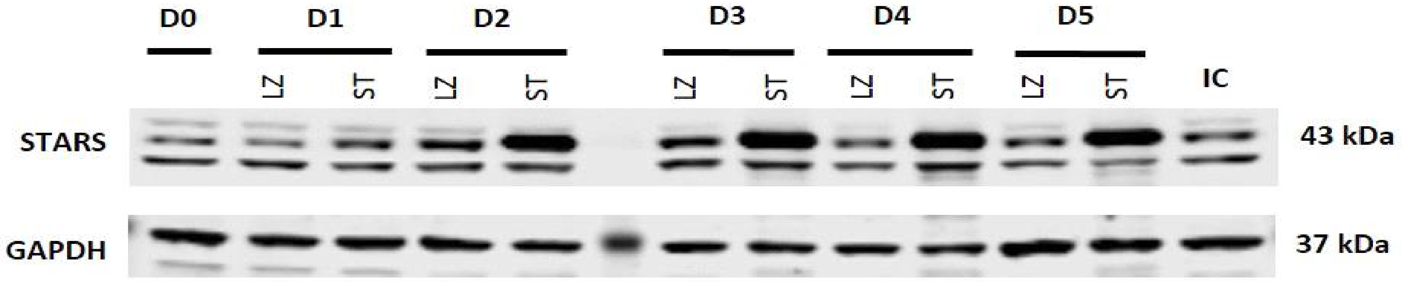

7G

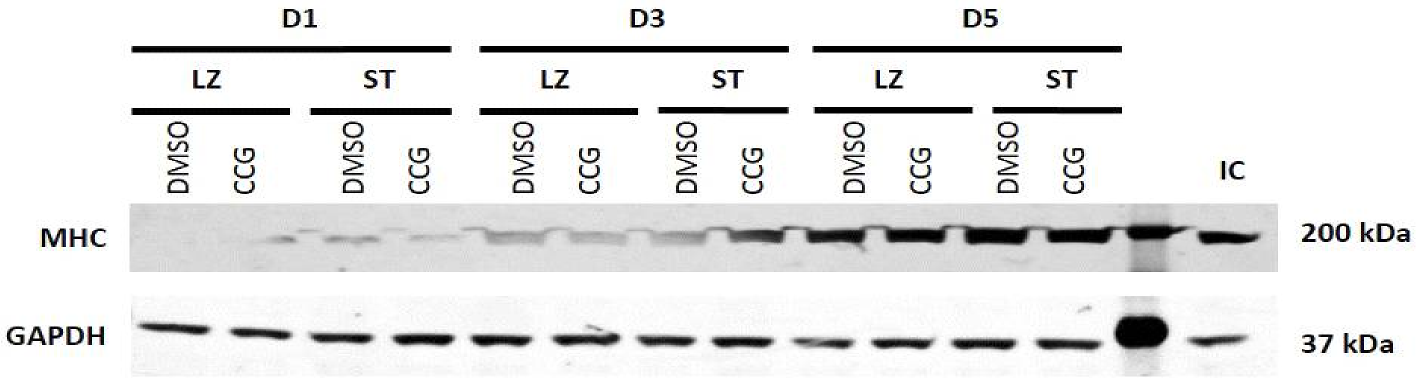

Supplement: Supplementary Figure 1 — Full western blot images of all immunoblot pictures presented in the manuscript. [file DataSheet1.pdf]
