# Supplementary material for: Overexpression of Striated Muscle Activator of Rho Signaling (STARS) Increases C2C12 Skeletal Muscle Cell Differentiation
Source: Front Physiol. 2016 Feb 8;7:7. doi: 10.3389/fphys.2016.00007 (PMC4745265; doi:10.3389/fphys.2016.00007)
Supplement: Supplementary Figure 2 — (A) STARS mRNA levels following STARS overexpression in C2C12 myoblasts. (B) STARS protein levels following STARS overexpression in C2C12 myoblasts. (C) 2H Enrichment expressed as a percentage of EM1 following deuterium incorporation assay. (D) Absorbance measured at 485 nm following a cytotoxicity assay in C2C12 myoblasts. (E) Absorbance measured at 485 nm following a cytotoxicity assay in C2C12 myotubes. (H) MRTF-A cellular localization following CCG-1234 treatment in serum-stimulated (20% FBS) and serum-free (SF) C2C12 myotubes at differentiation day 5. Immunolocalization of MRTF-A. Blue, DAPI staining; Red, MRTF-A; DMSO, dimethyl sulfoxide vehicle control; CCG-1423, SRF inhibitor. (G) MRTF-A intensity in the nucleus divided by total MRTF-A intensity in C2C12 myotubes at differentiation day 5. [file DataSheet2.PDF]

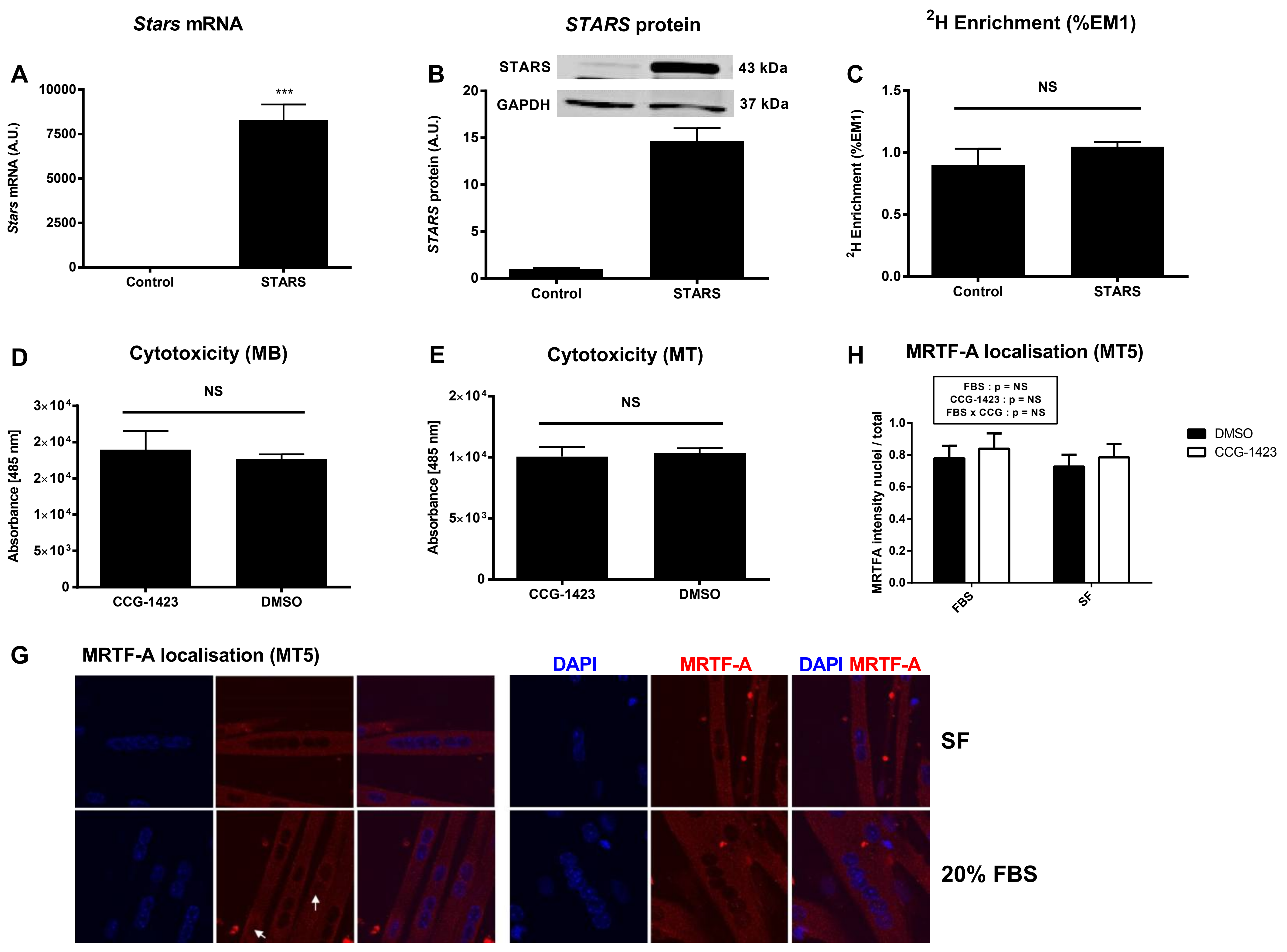

Stars mRNA
STARS protein
2H Enrichment (%EM1)
A
B
C
Stars mRNA (A.U.)
10000
7500
5000
2500
0
***
Control
STARS
STARS
43 kDa
GAPDH
37 kDa
STARS protein (A.U.)
20
15
10
5
0
Control
STARS
NS
2H Enrichment (%EM1)
1.5
1.0
0.5
0.0
Control
STARS
D
Cytotoxicity (MB)
NS
Absorbance [485 nm]
3×10^4
2×10^4
2×10^4
1×10^4
5×10^3
0
CCG-1423
DMSO
E
Cytotoxicity (MT)
NS
Absorbance [485 nm]
2×10^4
1×10^4
5×10^3
0
CCG-1423
DMSO
H
MRTF-A localisation (MT5)
FBS : p = NS
CCG-1423 : p = NS
FBS x CCG : p = NS
MRTFA intensity nuclei / total
1.0
0.8
0.6
0.4
0.2
0.0
FBS
SF
DMSO
CCG-1423
G
MRTF-A localisation (MT5)
DAPI
MRTF-A
DAPI MRTF-A
SF
20% FBS
